# A Saudi Woman with Ceftriaxone Induced Fixed Drug Eruption

**DOI:** 10.1155/2024/9975455

**Published:** 2024-03-15

**Authors:** Rehab Y. Al-Ansari, Leena Abdulrahman Almuhaish, Khaled Abdullah Hassan, Tawasoul Fadoul, Alexander Woodman

**Affiliations:** ^1^Adult Hematology Unit, Internal Medicine Department, King Fahd Military Medical Complex, Dhahran, Saudi Arabia; ^2^Dermatology Unit, Internal Medicine Department, King Fahd Military Medical Complex, Dhahran, Saudi Arabia; ^3^General Medicine Unit, Internal Medicine Department, King Fahd Military Medical Complex, Dhahran, Saudi Arabia; ^4^School of Health Sciences, University of Salford, Manchester, UK

## Abstract

**Background:**

A fixed drug eruption (FDE) is an immunological cutaneous adverse reaction, classified as a cutaneous adverse drug reaction (CADR) and characterized by well-defined lichenoid lesions that occur at the same site each time. Ceftriaxone is a third-generation antibiotic of cephalosporin antibiotics of the beta-lactam antibiotic family, which has typical *in vitro* activity against many Gram-negative aerobic bacteria. This is the first clinical case from Saudi Arabia and the fifth in the world to document a woman's experience with recurrent FDE after repeated ceftriaxone use. *Case Report*. A 25-year-old Saudi woman with a known case of sickle cell anemia (SCA) with a history of avascular necrosis of the right hip after replacement was hospitalized with a pain crisis triggered by an upper respiratory tract infection. The patient denied having a history of allergy previously. Due to fever, leukocytosis, and active follicular tonsillitis, ceftriaxone was started. However, a few hours later she developed lip edema and a fixed drug eruption measuring 7 × 11 cm on the left side of her back. The lesion reformed over a hyperpigmented lesion (4 × 8 cm) that the patient did not report upon initial examination. It turned out that this was due to the intravenous administration of ceftriaxone, a year ago in another hospital. An allergy to ceftriaxone was considered, and steroids and antihistamines were started. The case was labeled as ceftriaxone induced FDE.

**Conclusion:**

Ceftriaxone induced FDE is an uncommon type of allergic reaction that has been reported infrequently. Understanding this condition and the mechanism by which FDE becomes recurrent with the same previous fixed lesion is of great importance for both academic and future research purposes.

## 1. Introduction

A fixed drug eruption (FDE) is an immunological cutaneous adverse reaction, classified as a cutaneous adverse drug reaction (CADR) and characterized by well-defined lichenoid lesions that occur at the same site each time [1–3]. Exposed to a systemically administered drug, FDE is unique in repeated exposure to the same medication and lighting up the older lesions [4–6]. The FDE affects people of all ages, races, and sexes, although some rare studies favor the female sex, which predominates in the 3rd and 4th decades of life. When treatment is terminated, the skin lesions may resolve but usually result in long-term or even permanent pigmentation in the form of oval erythematous patches [7–9]. FDE accounts for 16–20% of all skin rashes, with the most common FDE-causing drugs falling into the following categories: antibiotics, nonsteroidal anti-inflammatory drugs, phenothiazines, antiepileptics, and sildenafil. Although drug provocation tests and patch tests applied to the affected area can be used in the diagnosis of FDE, the diagnosis is usually made clinically [7, 10].

Ceftriaxone is a third-generation antibiotic of cephalosporin antibiotics of the beta-lactam antibiotic family, which has typical *in vitro* activity against many Gram-negative aerobic bacteria. It is the only cephalosporin whose dose does not need to be adjusted in the presence of renal insufficiency unless there is an associated hepatic and renal dysfunction [11, 12]. Cephalosporins, including ceftriaxone, have low toxicity and are generally safe, with the most common adverse reactions being nausea, vomiting, anorexia, and abdominal pain. Less common adverse reactions include hypersensitivity reactions, drug-induced immune hemolytic anemia (DIIHA), disulfiram-like reaction, vitamin K deficiency, increased aminoglycoside nephrotoxicity, and pseudomembranous colitis [11–13]. Sensitivity to cephalosporins may occur due to the beta-lactam core, cephalosporin ring, or side chains, meaning there is a potential risk of cross-reactivity with many beta-lactam antibiotics. The fact that sensitivity is mainly related to the side chains allows many patients to tolerate various beta-lactam drugs and even cephalosporins, which allows the use of beta-lactam antibiotics. Therefore, allergy evaluation of these patients is necessary [14, 15]. This is the first clinical case from Saudi Arabia and the fifth in the world to document a woman's experience with recurrent FDE after repeated ceftriaxone use.

## 2. Patient Information

A 25-year-old Saudi woman with sickle cell anemia (SCA) and bilateral avascular necrosis of the hip, who had undergone a right hip replacement three years ago, received 5 mg folic acid tablets once daily, presented to the emergency department complaining of generalized body pain, and described as her usual pain crisis with sore throat and fever for one day. The patient had no contact with a sick patient/people and did not travel recently. The patient was admitted to the hematology department for a vaso-occlusive crisis with acute follicular tonsillitis. The patient denied a history of drug allergy or hypersensitivity reactions. Due to the high body temperature of 39°C and leukocytosis, analgesia was prescribed for SCA in the form of hydration and anesthesia (paracetamol, ibuprofen, and morphine). In addition to antibiotics, ceftriaxone of 2 g IV every 24 hours was prescribed.

### 2.1. Clinical Finding

On examination, blood pressure was 102/66 mm Hg, pulse was 110/min, respiratory rate was 20 breaths/min, and oxygen saturation was 99% in room air. There was no jaundice or cyanosis and no palpable cervical lymphadenopathy. The throat was clogged with a whitish patch on the left posterior tonsil and a pattern of follicles suggestive of follicular tonsillitis. The chest was clean with equal air intensity on both sides. The abdomen was soft, flaccid, without pain, and palpable organomegaly.

### 2.2. Diagnostic Assessment

Laboratory tests showed hemoglobin (Hgb) of 8.3 g/dl [12–16], mean corpuscular volume (MCV) of 86 *μ*l (80–94), mean corpuscular hemoglobin (MCH) of 32.5 pg (27–32), platelet (PLT) count of 353 × 10^3/*μ*l (140–450), and white blood cell (WBC) count of 18 × 10^3/*μ*l [4–11], with monocytosis of 1.2 × 10^3/*μ*l (0.2–0.8) and neutrophilia of 8 × 10^3/*μ*l (2–7.5). The G6PD screen was negative with C-reactive protein (CRP) of 34 mg/L and an erythrocyte sedimentation rate (ESR) of 27 mm/hr (0–20). Biochemistry results showed signs of active hemolysis with normal electrolytes and renal panel (Table 1). Peripheral blood smears showed sickle-shaped cells with some anisocytosis and poikilocytosis but no blast cells.

### 2.3. Therapeutic Intervention

**Figure 1:**
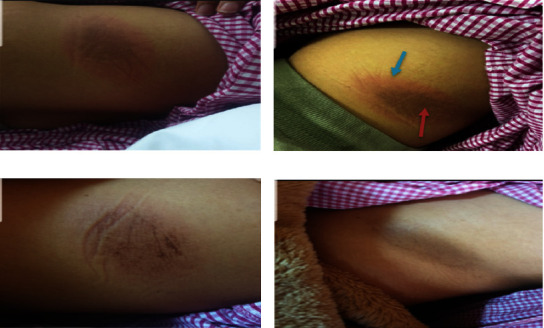
(a) First-day post ceftriaxone administration with a new hyperpigmented reddish lesion (7 × 11 cm) with a central old brownish lesion (4 × 8 cm), (b) same day with a closer view for new fixed drug eruption (blue arrow) and old central eruption (red arrow), (c) two days after the onset with gradual resolving of the new lesion with central brownish linear streaks, and (d) three weeks after onset, the lesion cleared leaving residual old hyperpigmentation.

On the same night and 4 hours after starting the antibiotic (ceftriaxone), the patient complained of an itchy skin lesion on her left side. Vital signs were within normal limits, without fever. On examination, a solitary well-defined erythematous oval patch with a brownish center, approximately 7 × 11 cm, was found on the left lateral side of the patient's back, which was formed on top of a previous hyperpigmented lesion (4 × 8 cm) (Figures 1(a) and 1(b)).

The patient had a history of the same lesion at the same site a year ago after being exposed to ceftriaxone at a different center. Angioedema of the upper lip was also noted. The patient denied the presence of shortness of breath (SOB) or dysphagia. There were no other rashes or itching elsewhere. Recurrence of the lesion at the same site on repeated administration of a particular drug, i.e., ceftriaxone, established the diagnosis of FDE. The administration of ceftriaxone was discontinued, and the patient was prescribed 2 stationary doses of hydrocortisone 100 mg intravenously, followed by 30 mg prednisolone with a rapid dose taper over the course of a week, as well as antihistamines.

### 2.4. Follow-Up and Outcomes

The antibiotic was replaced with azithromycin 2 tablets of 250 mg (total dose of 500 mg) 1 time per day. Betamethasone cream was administered twice daily and desloratadine once daily at bedtime. Of note, the eosinophil count measured after administration of standard doses of hydrocortisone and antihistamine was normal at 0.271 × 10^3/*μ*l (0–0.8), and immunoglobulin E (IgE) was not administered. Two days later, the lesion became pale and brownish linear stripes appeared, and as the surrounding erythema disappeared, the size became approximately 7 × 5 cm (Figure 1(c)). Angioedema completely resolved. The patient was instructed to continue applying betamethasone cream twice daily for 1 week. Three weeks after the onset of the lesion, the lesion disappeared (Figure 1(d)). As per the hospital's protocol, the patient was informed and provided additional information in the discharge chart that ceftriaxone and other cross-reacting drugs should not be taken and should inform the next attending physician.

## 3. Discussion

The onset of FDE is from 0 to 45 days, and the relapse duration varies from 1 to 20 years [2, 7, 16]. In this study, the case developed itchiness on top of the previous lesion at about 4 hours of exposure and one year after the first onset in the past, which are consistent with data from previous cases.

The ceftriaxone as the causative agent of the FDE found, in this case, was unique, as previous data informed only rare reported cases. Thus, Ozkaya et al. reported the first case of ceftriaxone induced FDE in a Turkish woman without cross-reactivity to other beta-lactam drugs [17]. Three more cases have been reported since 2008, two of them are males, all under the age of forty, along with a history of prior exposure to ceftriaxone [4, 18, 19]. The fourth case is reported by Mitre et al., with generalized bullous fixed drug eruption (GBFDE) as a form of FDE facilitated diagnosis [20]. However, concerns about the role of other comorbidities that may precipitate generalized bullous formation, such as renal failure and systemic lupus erythematosus, make the case scenario uncertain [20]. Thus, this case, described in a young woman from Saudi Arabia, is unique in which the patient developed swelling of the lips (angioedema) due to FDE, making this study a form of mixed ceftriaxone induced hypersensitivity reaction.

Treatment for FDE is mainly symptomatic relief. The first step is to stop the drug, followed by antihistamines, and topical corticosteroids if needed [7]. Additional treatments have also been reported, such as desensitization and the use of cyclosporine [7, 21]. In addition, the need for antibiotics is individual and depends on cross-reactivity with the pathogen. In this case, the change from ceftriaxone to azithromycin was without cross-reactivity. Finally, in this case, intravenous steroids were required due to the development of angioedema, which caused more serious concomitant reactions than simple local FDE.

The use of the Naranjo scale is recommended in future studies to help standardize the assessment of causality for all adverse drug reactions if allergic assessment of ceftriaxone reactions is not available [22, 23]. This is a method which can assess whether a causal relationship exists between an identified adverse clinical event and a drug by using a simple questionnaire to assign probability scores. Although the scale was developed for use in controlled trials and registration studies of new drugs rather than in routine clinical practice, it is widely used in daily practice [22, 23].

## 4. Conclusion

Ceftriaxone induced FDE is an uncommon type of allergic reaction that has been reported infrequently. Understanding this condition and the mechanism by which FDE becomes recurrent with the same previous fixed lesion is of great importance for both academic and future research purposes.

### 4.1. Limitations

Taking into consideration the rarity of the medical condition and the emergency situation, this report has some limitations that were considered retrospectively after the patient was treated and the report was documented. Shortness of breath can be a panic reaction. However, dyspnea and dysphagia also reflect the degree of allergy, indicating angioedema. After consultation with a dermatologist colleague who recommended topical steroids, the antihistamine was discontinued during the same appointment. In hindsight, we recognize that there was no need to cut back on the short course of steroids.

## Figures and Tables

**Table 1:** Chemistry laboratory results upon admission.

Name	Results	Reference range
Albumin	37	34–50 g/L
Alkaline phosphatase	70	50–136 U/L
ALT	26	16–36 U/L
AST	37	15–34 U/L
Conjugated bilirubin	12	0–3 *μ*mol/L
Bilirubin (total)	31	3–17 *μ*mol/L
LDH	403	85–227 U/L
GGT	26	15–85 U/L
Creatinine	42	62–115 *μ*mol/L
BUN	1.1	2.5–6.4 mmol/L
Sodium	141	136–145 mmol/L
Potassium	3.7	3.5–5.1 mmol/L
Calcium	2.17	2.12–2.52 mmol/L

LDH, lactic acid dehydrogenase; ALT, alanine aminotransferase; AST, aspartate aminotransferase; GGT, gamma-glutamyl transferase; BUN, blood urea nitrogen.
